# Concepts and definitions of healthy ageing: a systematic review and synthesis of theoretical models

**DOI:** 10.1016/j.eclinm.2022.101821

**Published:** 2023-01-12

**Authors:** Marilyne Menassa, Karien Stronks, Farnaz Khatmi, Zayne Milena Roa Díaz, Octavio Pano Espinola, Magda Gamba, Oche Adam Itodo, Chepkoech Buttia, Faina Wehrli, Beatrice Minder, Minerva Rivas Velarde, Oscar H. Franco

**Affiliations:** a Institute of Social and Preventive Medicine, University of Bern, Bern, Switzerland; b Graduate School for Health Sciences, University of Bern, Bern, Switzerland; c Department of Public and Occupational Health, Amsterdam University Medical Centers, University of Amsterdam, Amsterdam, the Netherlands; d Community Medicine Department, Tehran University of Medical Sciences, Tehran, Iran; e Navarra Institute for Health Research (IdiSNA), Pamplona, Spain; f Swiss Paraplegic Research, Nottwil, Switzerland; g Public Health and Primary Care Library, University Library of Bern, University of Bern, Bern, Switzerland; h Department of Radiology and Medical Informatics, Faculty of Medicine, University of Geneva, Geneva, Switzerland; i Department of Public Health, Julius Center for Health Science and Primary Care, UMC Utrecht, Utrecht University, Utrecht, the Netherlands

**Keywords:** Healthy ageing, Concept, Definition, Model, Dimension, Concept analysis

## Abstract

**Background:**

Healthy ageing (HA) has been defined using multiple approaches. We aim to produce a comprehensive overview and analysis of the theoretical models underpinning this concept and its associated normative terms and definitions.

**Methods:**

We conducted a systematic review of peer-reviewed HA models in Embase.com, Medline (Ovid), Cochrane CENTRAL, CINAHL, PsycINFO, and Web of Science until August 2022. Original theoretical papers, concept analyses, and reviews that proposed new models were included. Operational models/definitions, development psychology theories and mechanisms of ageing were excluded. We followed an iterative approach to extract the models’ characteristics and thematically analyze them based on the approach of Walker and Avant. The protocol was registered in PROSPERO (CRD42021238796).

**Findings:**

Out of 10,741 records, we included 59 papers comprising 65 models/definitions, published in English (1960–2022) from 16 countries in Europe, Asia, and America. Human ageing was described using 12 normative terms, mainly (models (%)): successful (34 (52%)), healthy (eight (12%)), well (five (8%)), and active (four (6%)). We identified intrinsic/extrinsic factors interacting throughout the life course, adaptive processes as attributes, and outcomes describing ageing patterns across objective and subjective dimensions (number of models/definitions): cognitive (62), psychological (53), physical (49), social (49), environmental (19), spiritual (16), economic (13), cultural (eight), political (six), and demographic (four) dimensions. Three types of models emerged: health-state outcomes (three), adaptations across the life course (31), or a combination of both (31). Two additional sub-classifications emphasized person-environment congruence and health promotion.

**Interpretation:**

HA conceptualizations highlight its multidimensionality and complexity that renders a monistic model/definition challenging. It has become evident that life long person-environment interactions, adaptations, environments, and health promotion/empowerment are essential for HA. Our model classification provides a basis for harmonizing terms and dimensions that can guide research and comparisons of empirical findings, and inform social and health policies enabling HA for various populations and contexts.

**Funding:**

MM, ZMRD, and OI are supported by the European Union’s Horizon 2020 Marie Skłodowska-Curie grant No 801076, and MM is also supported by the Swiss National Foundation grant No 189235.

**Research in context**

**Evidence before this study**

We conducted an umbrella review of healthy ageing concepts and definitions in pubmed and google scholar between October and December 2020 using the terms: healthy, successful, active, and optimal ageing combined with definition, model, concept, domain, construct, determinant, mechanism, or dimension. We included reviews and concept analyses of theoretical and operational models and definitions in social and medical gerontology, mechanisms of ageing, and determinants of healthy ageing until December 31, 2020. The findings showed a great heterogeneity in defining and operationalizing this concept and a gap on systematic reviews and analyses that comprehensively map out the theoretical literature on healthy ageing. This gap underlies the lack of clarity on the theoretical underpinnings from which operationalizations of healthy ageing emerged.

**Added value of this study**

By combining systematic review and concept analysis approaches in synthesizing theoretical models, this study comprehensively delineates and clarifies the concept of HA and its dimensions and explores whether a consensus is possible on a definition or model valid and applicable in multiple contexts.

**Implications of all the available evidence**

Bringing convergence and conceptual clarity to HA has wide implications in identifying gaps and improving the clinical and research applications of the concept in the context of existing operationalizations, in exploring the validity of existing indices and measures across the different dimensions of ageing, and in informing policies to enable HA measures and strategies for everyone.

## Introduction

The increase in life expectancy at birth and global demographic shift into an older age present unprecedented social and economic challenges to the modern world.^1^ This is of particular concern as this increase in life expectancy is often accompanied by increased years spent in ill health.^2^ This raises the question of how to promote healthy ageing (HA) in the population.

The WHO defines HA as maintaining a functional ability that enables individuals to meet their needs and contribute to society within their environment. However, many other definitions also exist. Past efforts in exploring HA show an explosion of normative terms, including active, resilient, and successful ageing, among many others, and a great heterogeneity in operationalizing conceptual models and definitions.^3^, ^4^, ^5^, ^6^, ^7^ Perhaps the difficulty in achieving conceptual clarity in these terms lies in the complexities of the network of biological mechanisms underlying the ageing process, the different meanings of HA for different populations and contexts, and the ongoing debate on the concept of health.^8^, ^9^, ^10^, ^11^, ^12^, ^13^, ^14^, ^15^ Aligning concepts constitutes a priority action toward shaping policies and optimizing HA with targets set by WHO for 2030.^2^

Understanding the theoretical grounds underpinning the operationalizations of HA is a requirement for two reasons: to advance empirical research on clear conceptual dimensions and outcomes across various populations and contexts and to consequently enable the implementation of evidence-based strategies targeting biological, demographic, social, psychological, and behavioural determinants of HA in these settings.^16^^,^^17^ This clarification has not proved to be simple. Several researchers have taken on the task of reviewing and analyzing theoretical models of HA and its associated terms.^18^, ^19^, ^20^, ^21^ Chapman for e.g. reflects on six frameworks of ageing well to construct a narrative around self-development that accompany life changes. In contrast, more recent reviews are focused on critiques of existing models, exploring researcher vs. older adult definitions and questions around the feasibility and desirability of HA.

To the best of our knowledge, none of the available reviews takes on the basic task of rethinking the foundations of HA theoretical models and aim to set a clear basis for future studies by combining systematic review and conceptual analysis methods. There is an ensuing need for a homogenized approach in understanding the use of terms referring to HA as well as mapping the characteristics of this concept. We aim to systematically review the literature on HA, analyze the theoretical models and definitions, explore the related normative terms and concepts, and produce a comprehensive thematic overview of what constitutes HA's dimensions, attributes, antecedents, and consequences.

## Methods

We conducted a systematic review and conceptual thematic analysis of HA models and definitions following the 24-step guide and PRISMA guidelines.^22^ The protocol is registered in PROSPERO under CRD42021238796, which was edited to accommodate the change in the reporting of findings to two separate papers because the results were too broad to be combined in one paper. The current paper includes theoretical models and subsequent efforts will consider operational and empirical models.

### Search strategy and selection criteria

A search strategy was developed with the help of experts, adapted and completed across Embase.com, Medline (Ovid), Cochrane CENTRAL, CINAHL, PsycINFO, and Web of Science until August 17, 2022. The main search terms were healthy, successful, active, robust, positive, optimal, well, and productive ageing combined with definition, construct, model, theory, concept, and dimension. The full search strategy per database is available in (Supplement 1).

We included published peer-reviewed original conceptual articles, concept analyses, and reviews only when these proposed new theoretical definitions or models with a normative description of ageing. We excluded empirical models from qualitative and quantitative studies, operational definitions and validations, studies on HA determinants and risk factors, and animal/experimental studies on mechanisms of ageing. Lifespan development and psychology theories that tackle ageing outside the context of HA were excluded. No limit on publication year or language was added.

We also conducted backward search of the reference lists of included papers and a forward citation search for full-text review to identify other eligible articles not identified in our initial search. When original papers of newly identified models from the reviews could not be found or were published in books, we included the earliest reference we could access that allowed the extraction of the model and noted this as a forward search paper.

Results were exported into EndnoteX8 for screening. Two reviewers independently screened the articles by title and abstract. When consensus could not be reached, a third independent reviewer was consulted. The full text was retrieved for all included articles available online and by contacting authors. Similarly, two reviewers screened full texts, and reasons for exclusion were noted. Non-english full-text papers were reviewed by team members who were fluent in the language.

### Data analysis

Data were extracted using a predesigned excel sheet piloted with key theoretical papers. Two reviewers independently extracted the main characteristics, including author, title, country, year, term, theoretical framework, definitions, dimensions, descriptions, antecedents, and consequences of the models. Information on empirical support was noted.

We followed an iterative approach to extract the characteristics of the models and thematically analyze them. This process was informed by the strategies of Walker and Avant for analyzing concepts According to this approach, a conceptual model is separated into its different constituting elements: the antecedents or what causes the concept, the consequences or the outcomes of the concept, and the attributes or the describing characteristics of the concept. We developed a glossary for attributes, antecedents, and consequences, and defined dimensions according to the Merriam-Webster dictionary^23^^,^^24^ (Table 1). The models and their descriptions were examined to determine the dimensions, attributes, antecedents, and consequences. To account for the heterogeneity in the use of terms, we adopted a comprehensive approach of overarching domains to specify dimensions (sometimes referred to in the models as constructs or domains), which was also informed by Ebert's multidimensional model and WHO's definition of health.^25^ A database of variables for the dimensions determined from the definitions/descriptions and outcomes was then collapsed into summary variables and classified as subjective or objective. The initially extracted variables for attributes, antecedents, and consequences were organized into inventories of descriptions/processes, determinants, and outcomes, respectively. These were then examined for relations and collapsed into broader summary themes, informing the types of the models based on commonalities/differences and dominant approach. Models with more than one approach were classified into more than one type. Because of the conceptual nature of the review, an evaluation of the risk of bias and quality assessment was not fit for purpose. Our rationale stems from the processes of theory and concept development described by Walker and Avant. The evaluation of a concept or theory occurs through its empirical validation in different populations/contexts, sometimes necessitating multiple revisions and validations to become practically useful. Furthermore, and to the best of our knowledge, an objective tool for theory and concept assessment in health outside empirical validation is not available.

**Table 1:** Glossary of terms and definitions.

(1) Dimensions terms^a^	Definition/synthesis approach	Eligible dimensions or domains from the included conceptual models
Dimensions	Dimensions are based on definitions, descriptions or attributes, and outcomes of the concept.	
Subjective	Dimensions that require individuals to evaluate their own healthy ageing subjectively	Spiritual, social, cultural, and psychological dimensions that require subjective opinion or evaluation
Objective	Dimensions that require the researchers to assess healthy ageing via defined objective criteria	Cognitive, physical, demographic, economic, environmental, political, and certain social, cultural, and psychological dimensions that require objective evaluation
Cognitive	Of, relating to, or involving conscious mental activities (such as thinking, understanding, learning, and remembering)	Memory, cognitive performance, cognitive and mental capacity, wisdom, requiring a judgment such as self-reflection, self-assessment etc.
Cultural	The beliefs, customs, arts, etc., of a particular society, group, place, or time	Habits, traditions
Demographic	The statistical characteristics of human populations	Age, ethnicity, gender
Economic	Of, relating to, or based on the production, distribution, and consumption of goods and services	Financial resources/income, retirement, professional status, economically productive activities
Environmental	The circumstances, objects, or conditions by which one is surrounded	Built and natural environment, technical/support, healthcare services, transportation
Physical	Of or relating to the body	Biological body function, structure, physiology, health, diseases, physical biological well-being, (instrumental) activities of daily living
Political	Of or relating to politics or government	Policy (vision), legal (Judiciary set of laws (formal law))
Psychological/behavioural	The science or study of the mind and behaviour	Mental health, coping, personality traits (self-efficacy), attitude, life satisfaction, subjective well-being, emotions, physical activity, lifestyle
Social	Relating to or involving activities in which people spend time talking to each other or doing enjoyable things with each other	Social environment, social network, community/civic involvement/volunteering, social provisions, social support, social role/activity in family or community
Spiritual	Things of a spiritual, ecclesiastical, or religious nature	Religious, personal values, meaning of life, life goals, dealing with death
**(2) Concept analysis terms**^b^		

Attributes	The list of characteristics that immediately call the concept to mind. Processes are considered attributes because they describe the interaction between components of a model and cannot be antecedents or consequences.	
Antecedents	Antecedents are those events or incidents that must occur or be in place prior to the occurrence of the concept. The antecedent must therefore exist and precede the consequence, and should not be used to define the attribute for the concept.	
Consequences	Consequences are those events or incidents that occur as a result of the occurrence of the concept, the outcomes of the concept. A cause–effect relationship exists between an antecedent, the determiner and a consequence, the determined. Both the antecedent and the consequence therefore exist as objective realities.	
Outcome/lifecourse approaches	Outcome models focus on the endpoint at one point in time such as occurrence of diseases, disabilities, or mortality instead of the life processes of ageing. Lifecourse models examine ageing as a developmental/changing/adaptation process across part/whole life span (life span theories, coping and adaptations, early ageing theories, transcendence theories.) leading to certain outcomes.	

a Based on the Merriam-Webster dictionary.

b Based on Walker and Avant definitions.

### Role of the funding source

No funding was sought for this paper. Marilyne Menassa, Zayne Milena Roa Díaz, and Oche Itodo are GlobalP3HS PhD Fellows whose projects have received funding from the European Union’s Horizon 2020 research and innovation programme under the Marie Skłodowska-Curie grant agreement No 801076, through the SSPH + Global PhD Fellowship Programme in Public Health Sciences (GlobalP3HS) of the Swiss School of Public Health. Marilyne Menassa is also co-funded by the Swiss National Foundation under grant number 189235 for LYRICA (Lifestyle Prevention of Cardiovascular Ageing) project. All authors had full access to the full data in the study and accept responsibility to submit for publication.

## Results

**Fig. 1:**
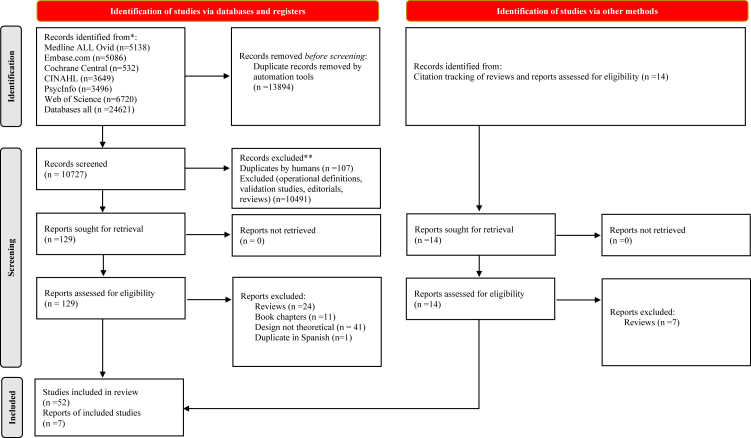
**PRISMA flowchart selection of studies**.

The search yielded 24,621 records, from which 10,727 were included for screening by title and abstract after de-duplication. After careful independent screening by two reviewers, 129 papers in English, Spanish, German, Farsi, and Dutch were selected for full-text screening in addition to 14 papers identified from citation tracking. Fifty-nine articles describing 65 models and definitions in English were eligible for inclusion (Fig. 1, Supplement 2). The papers were published between 1960 and 2022 in 16 countries in Europe, Asia, and America. Most originated from the USA (31), followed by Germany (six), the Netherlands (five), Mexico (three), Sweden (two), and Switzerland (two). Brazil, China, Canada, Italy, Jamaica, New Zealand, Philippines, Spain, Taiwan, and the UK contributed each with one article.

All 65 models adopted a theoretical approach in defining HA, while some included additional approaches. Twelve (18%) included practical outlook for guiding policy or interventions.^26^, ^27^, ^28^, ^29^, ^30^, ^31^, ^32^, ^33^, ^34^, ^35^, ^36^, ^37^ Six (9%) were empirically validated with original data,^38^, ^39^, ^40^, ^41^, ^42^, ^43^ and four definitions were derived from one common theory of successful ageing in one article.^44^ The authors described the models as theories or concepts which were either original or derived from concept analyses. Eight (12%) described theories that included the concept of successful and active ageing,^38^^,^^40^^,^^44^, ^45^, ^46^, ^47^, ^48^, ^49^ three of which were middle-range theories.^44^^,^^47^^,^^48^ Seven (11%) were generated from concept analyses: three on successful ageing, two on HA, and one on each of active and resilient ageing.^3^, ^4^, ^5^^,^^36^^,^^50^, ^51^, ^52^ All except 20 models (69%) were based on clear theoretical frameworks or were adapted from previous ones.^3^^,^^4^^,^^37^^,^^41^^,^^43^^,^^52^, ^53^, ^54^, ^55^, ^56^, ^57^, ^58^, ^59^, ^60^, ^61^, ^62^, ^63^, ^64^

In the 65 models, there were 12 terms used to refer to or evaluate human ageing: active, adjustment, well, compression of morbidity, graceful, healthy, sustainable, positive, productive, resilient, optimal, and successful. Successful ageing was the most widely used (number of models (%)): (34 (52%)), followed by HA (eight (12%)), ageing well (five (8%)), and active ageing (four (6%)), while the remaining 14 models used the other terms.

Most (62 models (95%)) included alife course approach to ageing, except for three (5%) that focused on specific outcomes in a single period.^41^^,^^57^^,^^65^ Fifteen (23%) were developed for specific populations or contexts, out of which: five for productive ageing or ageing at work (33%),^29^^,^^31^^,^^64^^,^^66^^,^^67^ three for people who are Lesbian, Gay or Bisexual (20%),^46^^,^^52^^,^^65^ two for people with HIV (13%),^33^^,^^35^ two for Chinese and/or American populations (13%),^29^^,^^68^ one for people with disabilities (7%),^30^ one for Low- Middle Income Countries (LMICs) (7%),^69^ and one for child health in HA (7%).^63^

*Conceptual dimensions*: The number of dimensions ranged between two (eight models (12%)), three (11 models (17%)), four (19 models (29%)), five (16 models (25%)), six (five models (8%)), seven (three models (5%)), eight (one model (2%)) and nine (two models (3%)). Among these, the most frequently included dimension (number of models) was cognitive (62), followed by psychological (53), physical (49), social (49), environmental (19), spiritual (16), economic (13), cultural (eight), political (six), and demographic (four). Most models included both objective and subjective dimensions (57), with cognitive dimension common to all except two models and one of the definitions derived from the activity theory,^44^^,^^66^^,^^70^ while eight models included only objective dimensions having physical and cognitive dimensions in common (Table 2, Supplement 3).

**Table 2:** Characteristics of included studies, models, and definitions.

Author	Year	Country of publication (corresponding author)	Language	Ageing term defined/modelled	Type and name of the concept model/framework, definition, or theory	Theoretical framework used or other models adapted or integrated	Paradigm (theoretical philosophical, applied outlook)	Definition or description of the concept/theoretical model (quoted from original text)	Lifecourse or outcome	Study type (original, secondary source (forward search), concept analysis)
Cumming et al.^38^	1960	USA	English	Theory of ageing	The disengagement theory	Theory	Theoretical philosophical (empirically supported by original data)	Successful aging means the acceptance and the desire for a process of disengagement from active life.	Lifecourse	Original (Forward Search)
Havighurst^44^	1961	USA	English	Successful ageing (4 definitions)	The activity theory	Middle-range theory	Theoretical philosophical	Successful aging means the maintenance as far and as long as possible of the activities and attitudes of middle age.	Lifecourse	Secondary (Original source could not be accessed) (4 definitions)
				Successful ageing	1st definition of the concept derived from the theory	The Activity Theory	Definition	One way of defining successful aging is to say that it consists of a way of life that is regarded by the society as appropriate for older people.	Lifecourse	
				Successful ageing	2nd definition of the concept derived from the theory	The Activity Theory	Definition	Successful aging may be defined as maintenance of the level and range of activities that characterize a person in his prime of life with a minimum downward adjustment.	Lifecourse	
				Successful ageing	3rd definition of the concept derived from the theory	The Activity Theory	Definition	Successful aging may be defined as a condition in which a person feels satisfied with his finances, family, friends, work, clubs, and church activity.	Lifecourse	
				Successful ageing	4th definition of the concept derived from the theory	The Activity Theory	Definition	This method assumes that a person who is aging successfully feels satisfaction with his present and his past life and asks him as skillfully as possible to report on his feelings about his life.	Lifecourse	
Loeb et al.^53^	1966	USA	English	Adjustment in ageing	Framework for adjustment in ageing		Theoretical philosophical	A congruence between organization of social space, living space, and time perspective leads to successful aging or high morale in old age.	Lifecourse	Original (Forward Search)
Havinghurst^55^	1968	USA	English	Successful ageing	Successful ageing definition		Theoretical philosophical	Successful ageing consists of successful adaptation. The actual forms of adaptation that result can be called patterns of aging. A pattern of aging is a coherent complex of behavior, including social interaction and use of free time, achieved by an individual through the interaction of his personality with his physical organism and with his social setting.	Lifecourse	Original
Gubrium et al.^45^	1972	USA	English	Ageing	Socioenvironmental theory of ageing	Theory	Theoretical philosophical	This approach assumes that the environment of action for the aged is two-sided and consequently is built on the interrelationship of two contextual dimensions: the first of these is social referring to the normative outcomes of social homogeneity, residential proximity, and local protectiveness. The second dimension will be referred to as the “individual context” indicating those activity resources such as health, solvency, and social support that influence behavior flexibility.	Lifecourse	Original (Forward Search)
Fries^71^	1980	USA	English	Compression of morbidity	Model of disease prevention	Survival and morbidity curves	Theoretical philosophical	Chronic diseases are approached with a strategy of postponement rather than cure. If the rate of progression is decreased, then the date of passage through the clinical threshold is postponed: if sufficiently postponed, the symptomatic threshold may not be crossed during a lifetime, and the disease is prevented.	Lifecourse	Original (Forward Search)
McClelland^39^	1982	USA	English	Adjustment to ageing	Path model	Aged Subculture and Activity Theory	Theoretical philosophical (empirically supported by original data)	The model tested here takes four central concepts: social activity, the quantity of an individual's interaction; social adequacy, the quality of an individual's social interaction as expressed in feelings of loneliness or its opposite; self-conception, the individual's image of his own worth as a person; and life satisfaction, or relative happiness with present circumstances in the context of one's lifetime experiences.	Lifecourse	Original
Ryff^72^	1989	USA	English	Successful ageing	An integrated model of personal development	Integration of theoretical perspectives: Life-span Developmental Theories, clinical theories of personal growth, and the mental health literature to elaborate the meaning of positive functioning in adulthood and old age	Theoretical philosophical	A model of wellbeing integrating self-acceptance, positive relations, autonomy, environmental mastery, purpose in life, and personal growth.	Lifecourse	Original
Friend^46^	1990	USA	English	Successful ageing	Theory of successful ageing in Lesbian and Gay	Social Construction Theory	Theoretical philosophical	This theory examines the process of successful ageing which is a result of the reconstruction of homosexuality as something positive within the following contexts: individual psychology, social and interpersonal dimensions; and legal and political advocacy. The model of identity formation contends that those who positively reconstruct the meaning of homosexuality develop resources which promote successful ageing.	Lifecourse	Original
Salthouse^56^	1991	USA	English	Ageing well	Concept model for accommodation		Theoretical philosophical	One possible strategy for dealing with cognitive decline is accommodation, in which deficit-revealing situations are avoided by altering the nature of the activities one performs.	Lifecourse	Original (3 models)
				Ageing well	Concept model for compensation		Theoretical philosophical	A second possible model for successful aging is compensation, in which there is an active or deliberate substitution of processes so that the same overall level of functioning is maintained through a modification in the way in which the activities are performed.	Lifecourse	
				Ageing well	Concept model for remediation		Theoretical philosophical	A third possible model for successful ageing is remediation in which some type of intervention is introduced to restore one's ability to prior level by improving the critical or deficient processes.	Lifecourse	
Staudinger et al.^73^	1993	Germany	English	Resilient and optimal ageing	Resilence and levels of reserve capacity model	Life-Span Theory and Selection, Optimization, and Compensation (SOC)	Theoretical philosophical	The model distinguishes among three levels of developmental functioning: pathological, normal, and optimal. Resilience focuses primarily on the maintenance and recovery of “normal” developmental functioning. Reserve capacity serves the attainment of further growth and “optimal” levels of functioning. In old age, less overall reserve capacity is available. Therefore, an increasing share needs to be allocated to the avoidance of negative or “pathological” outcomes.	Lifecourse	Original
Baltes et al.^54^	1996	Germany	English	Successful ageing	Metamodel of selective optimization with compensation (SOC)		Theoretical philosophical	It is a life-span model of psychological management that describes how individuals can deal with the dual-faced nature of human aging and the ubiquitous, age-related shift toward a less positive balance of gains and losses. SOC defines success as goal attainment and successful ageing as minimisation of losses and maximisation of gains. Using the notion of mastery and adaptation allows diverse specifications of the goals and its evaluation criteria depending on the specific theory tested.	Lifecourse	Secondary (Original source could not be accessed) (Forward Search)
Chang et al.^68^	1996	USA	English	Successful ageing	Theoretical model of successful ageing in the American and Chinese cultures	Diengagement and Activity Theories	Theoretical philosophical	The major thesis of the model is that congruence between the individual's age reference set and their activity level leads to high meaningful existence. Stated differently, cognitive dissonance is perceived as having a negative effect on meaningful existence.	Lifecourse	Original
Schulz et al.^74^	1996	USA	English	Successful ageing as successful development	Lifespan model of successful development: Model of developmental regulation across the life course	Lifecourse Theory of Control (integrated with SOC)	Theoretical philosophical	Successful aging includes the development and maintenance of primary control throughout the life course. The successful life course is achieved when selection and compensation processes serve to maximize the primary control of the individual over the life course. These goal cycles are composed of goal selection, goal engagement, and goal disengagement.	Lifecourse	Original
Rowe et al.^57^	1997	USA	English	Successful ageing	Model of successful ageing		Theoretical philosophical	We define successful aging as including three main components: low probability of disease and disease-related disability, high cognitive and physical functional capacity, and active engagement with life.	Outcome	Original
Steverink et al.^75^	1998	The Netherlands	English	Successful ageing	Model of successful ageing	Theory of Social Production Functions (SPF) (integrates SOC with Theory of Goals)	Theoretical philosophical	A model of successful ageing that shows how ageing individuals behave and adapt in the face of changing resources and constraints, and when this behaviour will be (un)successful. The process of ageing can be characterised as a changing balance between gains and losses (in resources), in which losses will increasingly outweigh gains. The essence of the model is the proactive individual, having resources to substitute, and being able to maintain wellbeing over the life-span even in the face of loss. Successful ageing is a patterned change in resources and goals.	Lifecourse	Original
Carstensen et al.^40^	1999	USA	English	Ageing	Socioemotional Selectivity Theory	Theory	Theoretical philosophical (empirically supported by original data)	Socioemotional selectivity theory addresses the role of time in predicting the goals that people pursue and the social partners they seek to fulfill them.	Lifecourse	Original (Forward Search)
Torres^70^	1999	Sweden	English	Successful ageing	Culturally-relevant theoretical framework	Kluckhohns Model of Value Orientations	Theoretical philosophical	It represents the relations between the foundations of value orientations, the value orientations themselves, and the conceptualisation of successful ageing that different cultures hold.	Lifecourse	Original
Godfrey^32^	2001	UK	English	Successful ageing	Dynamic sociocultural model of successful ageing	Adapted from SOC Baltes with culture component for prevention	Theoretical philosophical/Applied policy outlook	The dynamic model of successful ageing offers a theoretical framework for understanding and developing preventive services and strategies for older people in social care.	Lifecourse	Original
Kahana et al.^33^	2001	USA	English	Successful ageing	Successful aging: model of preventive-corrective proactivity (PCP) in people with HIV	Adapted from: Kahana, E. & Kahana, B., “Conceptual and empirical advances in understanding aging well through proactive adaptation.	Theoretical philosophical/Applied outlook	The PCP model is anchored in the stress paradigm. In defining successful aging, our model provides flexibility so as to allow for processes or outcomes alternatively serving as hallmarks of success. One might define success to be based on engaging in preventive or corrective adaptations, even if in the end those adaptations are insufficient to counteract the ill effects of stressors.	Lifecourse	Secondary (Original source could not be accessed)
Crowther et al.^76^	2002	USA	English	Successful ageing	Enhanced model of successful ageing	Adapted from Rowe and Kahn theoretical framework	Theoretical philosophical	We maintain, with our broadened Rowe and Kahn model, that ageing is multifaceted and consists of interdependent biological, psychological, social, and spiritual processes.	Lifecourse	Original
Flood^3^	2002	USA	English	Successful ageing	Concept model and definition		Theoretical philosophical	Based on this conceptual definition, successful ageing is based on three essential foundational elements upon which higher level processes build: functional status, spirituality, and gerotranscendence. Conceptual definition of successful ageing as an individuals perception of a favorable outcome in adapting to the cumulative physiologic and functional alterations associated with the passage of time, while experiencing spiritual connectedness, and a sense of meaning and purpose in life (Flood 2005).	Lifecourse	Concept Analysis
Kalache et al.^26^	2003	Switzerland	English	Active ageing	Policy framework	WHO definition of Active Ageing	Theoretical philosophical/Applied policy outlook	Active ageing is the process of optimizing opportunities for health, participation, and security in order to enhance quality of life as people age. The word Active refers to continuing participation in social, economic, cultural, spiritual, and civic affairs, not just in the labour force. Health refers to physical, psychological, and social well-being. Attaining the goal of active ageing will require intersectoral action in addition to health and social services including education, employment labour, finance, social security, housing, transportation, justice, rural, urban development.	Lifecourse	Original
Flood^47^	2005	USA	English	Successful ageing	Theory illustrated by a model	Middle-Range Theory based on Roy Adaptation Model and Tornstams sociological Theory of Gerotranscendence	Theoretical philosophical	Successful ageing assumptions are: 1. Ageing is a progressive process of simple to increasingly complex adaptation. 2. Ageing may be successful or unsuccessful, depending upon where a person is along the continuum of progression from simple to more complex adaptation and minimal to extensive use of coping processes. 3. Successful aging is influenced by the aging person's choices. 4. The self is not ageless. Ageing people undergo changes which uniquely characterize their beliefs and perspectives as different from those young adults.	Lifecourse	Original
Hansen-Kyle^4^	2005	USA	English	Healthy ageing	Definition illustrated by a model of healthy ageing		Theoretical philosophical	Healthy ageing is the process of slowing down physically and cognitively, while resiliently adapting and compensating in order to optimally function and participate in all areas of ones life (physical, cognitive, social, spiritual)	Lifecourse	Concept Analysis
Chapman^58^	2006	Canada	English	Ageing well	Materialist perspective to ageing well		Theoretical philosophical	Ageing well may be understood as a process in which older adults construct and re-construct a sense of self relative to changing levels of resources and activity amid later-life events and transitions. Through a new materialist lens, studying later–life relationships with special things is one way to study this identity construction.	Lifecourse	Original
Kanning et al.^59^	2008	Germany	English	Successful ageing	Bio-psychosocial (heuristic) model of successful ageing		Theoretical philosophical	The bio-psychosocial model explains that the chance to enhance Subjective Well-Being (SWB) is restricted by personal dispositions (e.g., physiological constitution, psychological factors) and social–structural constraints (e.g., predominant stereotype of aging, facilities especially for target groups).	Lifecourse	Original
Young et al.^61^	2009	USA	English	Successful ageing	Definition and conceptual framework		Theoretical philosophical	A state wherein an individual is able to invoke adaptive psychological and social mechanisms to compensate for physiological limitations to achieve a sense of well-being, high self-assessed quality of life, and a sense of personal fulfillment even in the context of illness and disability.	Lifecourse	Original
Leipold et al.^77^	2009	Germany	English	Successful ageing	An integrative model of coping, resilience, and development as a theoretical access to successful ageing	A developmental theoretical framework for coping processes: the Dual-Process Model of Developmental Regulation (Brandstadter 2006)	Theoretical philosophical	Development consists of successfully overcoming life problems and successful coping can be recognized exactly where overcoming future problems (i.e., development) remains possible, if not easier. Resilience as a phenomenon is defined by the success (positive developmental outcomes) of the (coping) processes involved (given the circumstances). Resilience may be viewed as a bridging concept between coping and development, not because it represents a missing link in any moderating or causal sense. The phenomenon is the “phenotypical” developmental stability of an individual living under adverse developmental conditions.	Lifecourse	Original
Williamson et al.^60^	2009	USA	English	Successful ageing	The activity restriction model of depressed affect		Theoretical philosophical	According to the Activity Restriction Model of Depressed Affect, to age successfully is to maintain physical and cognitive functioning via engagement in personally meaningful activities. Major life stressors lead to poorer mental health outcomes because they disrupt normal, valued activities. In other words, activity restriction mediates the association between stress and mental health.	Lifecourse	Original
Potempa et al.^27^	2010	USA	English	Healthy ageing	The Healthy Ageing Model: a practical model of health behaviour change for older adults	Theoretical Models of Health Behaviour Change	Theoretical philosophical/Applied outlook	The concept described here represents a model of health promotion for an ageing population, a model focused on supporting positive health behaviour changes in ageing adults. The model combines tested methodologies and applies established theoretical models of health behaviour change in a population of ageing individuals in varying states of health and illness. The Healthy Ageing Model is characterised by four elements: (1) a client-centered perspective, (2) a goal-driven approach, (3) an individualised ‘‘coaching’’ strategy of health behaviour change, and, (4) recognition of the importance of the broader health context in which clients live, described in the model as one's ‘‘Personal Health System.’’	Lifecourse	Original
Pruchno et al.^41^	2010	USA	English	Successful ageing	Conceptual model		Theoretical philosophical (empirically supported by original data)	We define successful aging as having both an objective and a subjective component. The objective component includes having few chronic diseases, ample functional ability, and little or no pain. The subjective component is an evaluation that individuals make of their own aging experience at one point in time. It includes how well they are aging, how successful their aging experience is, and the extent to which they rate their current life as positive.	Outcome	Original
Hill^28^	2011	USA	English	Positive ageing	Positive ageing strategy framework	Psychological Adaptation Framework	Theoretical philosophical/Applied outlook	Positive ageing is descriptive of psychological adaptation to the inevitable consequences of late-life decline. A positive aging approach to coping is captured in the ability to recruit latent potentiality (or psychological reserve capacity) and to respond flexibly in age-related transitions, to engage affirmative decision making processes, and to cultivate an optimistic view by reframing the deteriorative processes of aging in such a way that preserves life satisfaction.	Lifecourse	Original
Schroots^78^	2012	The Netherlands	English	Active ageing	The Janus model of life course dynamics applied to active ageing	The Janus Model of Life Course Dynamics (mathematical)	Theoretical philosophical	On the basis of the “butterfly” metaphor for development and ageing, the Janus dynamic systems model offers a quite satisfactory account of the life-course dynamics of simple and more complex growth and decline functions and is characterized by three principles: transition, peak capacity, and peak time. These principles might be applied in disentangling the underlying mechanisms of diverse aging trajectories.	Lifecourse	Original
Villar^79^	2012	Spain	English	Successful ageing	Developmental model of generativity in old age	Generativity	Theoretical philosophical	Successful ageing includes the ability to engage efficiently in adaptive processes so as to achieve meaningful goals, such as generative ones. Successful ageing forms part of a developmental framework which includes, in addition to growth, the maintenance of desirable states and the regulation of losses. Generativity in old age is a developmental model that is optimistic and which highlights the gains which can be made in older age.	Lifecourse	Original
Wahl et al.^80^	2012	Germany	English	Ageing well	Conceptual model of belonging and agency, ageing well, and the environment	Lawton's Ecology Theory of Ageing	Theoretical philosophical	We define ageing well as maintaining the highest autonomy, well-being, and preservation of one's self and identity as possible, even in the face of severe competence loss. Our model posits that the interaction of belonging and agency unfolds within a life-course perspective, with the processes of belonging increasing in importance as people enter old age, whereas the relevance of processes of agency decreases.	Lifecourse	Original
McCarthy et al.^50^	2013	USA	English	Successful ageing	Conceptual model of transcendence in maturation and ageing	Gerotranscendence and Self-Transcendence	Theoretical philosophical	Transcendence is a developmental process resulting in a shift in perspective from a rational, materialistic view to a wider world view, characterized by broadened personal boundaries within interpersonal, intrapersonal, transpersonal, and temporal dimensions. Five domains of transcendence in the model: relationships, creativity, contemplation, introspection, spirituality. The model suggests directionality among its components such that activities based on antecedents of self-transcendence within one domain might be expected to promote those attributes of self-transcendence within the same domain.	Lifecourse	Concept Analysis
Peng et al.^29^	2013	China	English	Productive ageing	Conceptual framework (China)	Relationship between Active Ageing, Social Participation, and Productive Ageing	Theoretical philosophical/Applied policy outlook	Under the premise of their willingness and capabilities, and on the basis of equal opportunities, older adults directly or indirectly participating in activities that are beneficial for personal and social development, in order to achieve the ultimate goal of harmonious development of personal and social values.	Lifecourse	Original
Wang et al.^62^	2013	Taiwan	English	Graceful ageing	Graceful ageing model (GAM) and lifespan development model		Theoretical philosophical	GAM should be focused not only on the criteria of positive outcomes but also on the dynamic processes of a successful ageing as well. Besides, the criteria of graceful ageing from the perspectives of the universal standard and the cultural/historical/individual specific are also necessary to be addressed comprehensively. The proposed model includes the three conceptions: criteria-based as goals to achieve, process-based for the dynamics of the ageing process, and the proactive coping to facilitate the ageing process to reach the criteria, as an action plan.	Lifecourse	Original
Felix et al.^63^	2014	The Netherlands	English	Child health in healthy ageing	Conceptual framework of child health in healthy ageing		Theoretical philosophical	A dynamic state, not merely the absence of disease or disability, but also adequate resilience that permits optimal physical, mental, and social functioning, and optimal quality of life, in order to achieve full potential and to become an independent, functional, and social individual.	Lifecourse	Original
Hicks et al.^51^	2014	USA	English	Resilient ageing	Concept definition and model	Rodgers's Evolutionary Method for evolving concepts and Antonovsky's (1979) Theory of Salutogenesis	Theoretical philosophical	Resilient aging is a process an older person endures beyond physical, psychosocial or cognitive adversity, through protective factors that influence of coping, hardiness, and self-concept, in the person's quest towards quality of life. (*The pathway in the model is depicted as a linear model, making it impossible to bypass the resilient ageing core. It is proposed that all older persons possess to some degree (i.e. positive or negative) the attributes of the resilient ageing core, which would ultimately affect their quality of life.*)	Lifecourse	Concept Analysis
Fowler et al.^42^	2015	New Zealand	English	Successful ageing	The Communicative Ecology Model of Successful ageing (CEMSA)	The Theory of Motivated Information Management	Theoretical philosophical (empirically supported by original data)	CEMSA is predicated on the belief that individuals have agency over the aging process and, through communicative practices, construct ecologies within which they are able to age more successfully. The CEMSA posits that uncertainty about aging prompts both emotional and communicative responses, and that these communicative responses create ecologies, or “ageing spaces” within which people have greater (or reduced) potential to age well.	Lifecourse	Original
Halkitis et al.^65^	2015	USA	English	Healthy ageing	Multi-level ecosocial conceptual model/framework for studying the health of ageing gay men	The Social Ecological Model of Health (Bronfennbrenner, 1986), the Theory of Syndemic Production (Singer, 2009) and the Behavioral Model of Health Service Utilization (Anderson, 1968)	Theoretical philosophical	A comprehensive framework developed around two sets of health-related outcomes: the health states (physical/mental/neurocognitive, and sexual health) and the healthcare utilization (defined as need, access, usage, and satisfaction) of older gay men.	Outcome	Original
Nilsson et al.^34^	2015	Sweden	English	Mindful sustainable ageing	Concept development of Mindful Sustainable Ageing (MSA)	4 theories: Activity, Disengagement, Gerotranscendence and Successful Ageing	Theoretical philosophical/Applied outlook	MSA integrates the theories of activity, disengagement, successful aging, and gerotranscendence, and elaborates these by emphasizing mindfulness practice as a vital part of a sustainable healthful aging.	Lifecourse	Original
Zacher^64^	2015	The Netherlands	English	Successful ageing at work	Theoretical framework of successful ageing at work (and working definition)		Theoretical philosophical	The theoretical framework includes five broad categories of work outcomes that are relevant and important to employees and organizations: work motivation, job performance, turnover and job search behavior, job attitudes, and occupational health and well-being. Working definition: Successful aging at work involves a comparison of employees' intraindividual age-related trajectories of a work outcome over time and across the working life span with other employees' age-related trajectories of the same outcome. (conceptualized successful ageing at work from a comparative perspective as positive deviations from the average intra-individual age-related trajectory of certain work outcomes)	Lifecourse	Original
Zolnikov^69^	2015	USA	English	Successful ageing model in LMICs	Conceptual model	Adapted from Rowe and Kahn	Theoretical philosophical	The new successful aging model focuses on eliminating likely hazards within the environment and aims to promote health knowledge and accessible health care, thereby reducing negative outcomes and allowing a higher level of physical and mental functioning.	Lifecourse	Original
Caceres et al.^52^	2016	USA	English	Successful ageing	Concept definition and model in LGB		Theoretical philosophical	Successful ageing in lesbian, gay and bisexual older people is defined as a subjective and multifactorial concept that is characterised by support from families of origin/families of choice, access to lesbian, gay, and bisexual-friendly services and the development of crisis competence skills which impact the ageing experience of LGB individuals.	Lifecourse	Concept Analysis
Udo^5^	2016	Jamaica	English	Active ageing	Concept model	Based on the WHO Active Ageing Framework	Theoretical philosophical	Active ageing is defined as “the process of optimizing opportunities for health, participation and security in order to enhance quality of life as people age” WHO 2002 policy framework	Lifecourse	Concept Analysis
Morrow-Howell et al.^66^	2017	USA	English	Productive engagement	Conceptual framework: a stock and flow diagram of productive engagement in later life	Ecological Systems Theory	Theoretical philosophical	The stock and flow diagram contains many feedback loops, from simple to complex, and all cannot be identified in a simple and clear fashion. Productive activity of older adults as workers, volunteers, and caregivers is at the center of the model. The model includes several stocks, including human and social capital of older adults, family resources, capacity of organizations to fulfill their purposes, programs and policies to support productive engagement of older adults, and societal attitudes and expectations about older adults. These stocks are all part of a complex system of feedback loops that determine the level of productive activity of older adults.	Lifecourse	Original
Salazar et al.^48^	2017	Mexico	English	Active Ageing Theory	Model of coping and adaptation with active ageing	Middle-Range Theory (MRT) containing the Active Ageing concept, based on Roy's Adaptation Model	Theoretical philosophical	Active aging represents general adaptation. For purposes of this proposal, active aging refers to those older adults who are physically independent, who interact with their environment, who are cognitively alert, who are free of symptoms of depression, and whose health is perceived as good or excellent despite having chronic diseases. The key concepts within the MRT are chronic diseases, hope, healthy habits, coping with aging, voluntary work, social support, and active aging. The links between concepts and the adaptation model are shown following the substruction model. The horizontal and vertical relationships are schematized from the conceptual model to the middle-range theory.	Lifecourse	Original
Tesch Romer et al.^30^	2017	Germany	English	Successful ageing	Propositions towards conceptualizing successful ageing as both ageing in good health and ageing with care needs	Rowe and Kahn	Theoretical philosophical/Applied outlook	The traditional concept of successful aging expanded to capture desirable living situations (autonomy, well-being) and to consider effective strategies and resources for aging in good health and aging with disability and care needs (individual, environmental, and care related strategies and resources).	Lifecourse	Original
Mendoza-Nunez et al.^43^	2019	Mexico	English	Healthy ageing	Community model of healthy ageing		Theoretical philosophical (empirically supported by original data)	The central objective of the model is to achieve an empowerment of older adults so that they actively and co-participate in and develop a healthy aging program, for which active ageing, education, the exercise of citizenship, resilience and generativity will allow the adoption of healthy lifestyles and behaviors that promote commitment (adherence) to the prevention and control of chronic diseases; it is about maintaining, prolonging and recovering physical, mental and social functionality, subjective well-being, life satisfaction and the ability to propose and develop life plans for the future: “the process that allows older people to adopt or strengthen healthy lifestyles, through selfcare, mutual help and self-management strategies, using optimally formal and informal social support networks, in order to maintain, prolong and recover physical, mental and social functioning, in order to achieve their maximum welfare, health, and quality of life, always within the specific sociocultural context”	Lifecourse	Original
Vance et al.^35^	2019	USA	English	Successful ageing	Bio-psychosocial model of successful ageing with HIV	Baltes and Baltes' SOC model as the framework for successful aging with HIV	Theoretical philosophical/Applied outlook	Successful aging refers to the ability to have a good perceived quality of life and well-being as one transitions through the advanced stages of life.	Lifecourse	Original
Kooij et al.^31^	2020	The Netherlands	English	Successful ageing	Process model for successful ageing at work	Motivational Theory of Lifespan Development	Theoretical philosophical/Applied outlook	We define successful aging at work as the proactive maintenance of, or adaptive recovery (from decline) to, high levels of ability and motivation to continue working among older workers.	Lifecourse	Original
Freund et al.^81^	2021	Switzerland	English	Healthy ageing	Heuristic model of motivation and healthy aging	WHO definition of Healthy Ageing and motivation science	Theoretical philosophical	Three-levels models: Goals (central in the model) are dynamic constructs that develop and change over time. The processes (next circle in the model) by which goals “come to life” and exert their influence on people's lives, namely goal setting, goal pursuit, and goal disengagement. The outer circles represent the contexts and environments in which people are situated.	Lifecourse	Original
Cardoso et al.^36^	2021	Brazil	English	Healthy ageing	Healthy ageing promotion model	Nola Pender’s theory of health promotion and Walker and Avant concept analysis	Theoretical philosophical/Applied outlook	The Healthy Ageing Promotion Model (HAPM) aims to assist nurses in understanding factors that influence healthy behaviors from the biopsychosocial context. When applying the concept of healthy aging in the HAPM diagram, it is understood to have produced a structure capable of guiding the operationalization of NP, enabling nurses to apply a theoretical model in nursing actions aimed at promoting healthy aging.	Lifecourse	Concept Analysis
Mastropietro et al.^37^	2021	Italy	English	Healthy ageing	NESTORE Multi-domain model of healthy ageing		Theoretical philosophical/Applied outlook	The NESTORE model was intended to provide a structured knowledge formalization to provide a simplified pool of information for (1) the characterization of the older adults, (2) the personalization of the coaching plans, and (3) the implementation of an effective IoT system.	Lifecourse	Secondary source (Original source could not be accessed) (Forward search)
Feliciano et al.^49^	2022	Philippines	English	Successful ageing	Ageing-related Resiliency Theory	Theory	Theoretical philosophical	In acknowledging the natural decline of essential processes, older adults respond to adapt and use coping techniques and resources to achieve and enhance resiliency that impacts optimism—aiding to age successfully (Aging-related Resiliency Theory). This theory hypothesizes that with advancing age, older adults assume to respond, accept, cope, and recover from life challenging experiences that hasten the capacity to resist over time which impacts optimistic outlook to age successfully	Lifecourse	Original
Uribe^67^	2022	Mexico	English	Productive ageing	Ecological model for productive ageing	Bronfenbrenner's human ecology model	Theoretical philosophical	According to Bronfenbrenuer's model, the environments in which people are immersed can be best represented using concentric structures named micro-meso-exo, macro, and chronosystems. Each of these systems affects individuals in different ways. According to the model, human development describes the processes by which individuals acquire a differentiated and valid understanding of their ecological environment. Aging can be considered as another stage in human development which is not only characterized by chronological age but by biographical and contextual factors including working late in life.	Lifecourse	Original

**Fig. 2:**
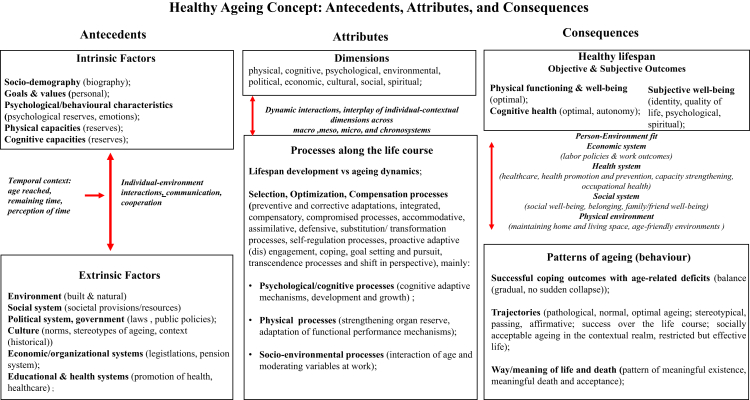
**Healthy ageing concept: antecedents, attributes, and consequences**.

*Antecedents*, *attributes*, *and consequences*: Our thematic concept analysis of the models yielded a list of antecedents, attributes, and consequences, summarized below (Fig. 2, Supplement 4):

*Antecedents* were classified into 11 themes of which five were intrinsic factors of individuals and six were extrinsic factors of the built and social environments and systems in which individuals age. The models represented dynamic interactions of these two groups of factors impacted by age and changing perceptions of the remaining time along the life course. Intrinsic factors included socio-demography and biography, personal goals and values, psychological and behavioural, physical, and cognitive capacities that define reserves, emotions, personalities, health, and life narratives. Extrinsic factors included the contextual provisions and resources reflected in the built and natural environments, and social, economic, political, health, cultural, and educational systems.

*Attributes* in the models were described by adaptative and development processes to lifelong changes based on dynamic interactions between individual and contextual life dimensions (physical, cognitive, psychological, environmental, political, cultural, social, economic, spiritual) and systems (macro, meso, micro, and chronosystems). These *attributes* include: selection, optimization, and compensation, coping, transformative adaptations, self-regulation, and transcendence processes across dimensions.

*Consequences* are the outcomes of the concept that are determined by the antecedents. They reflect the level of person-environment fit or congruence that is manifested by the individual's health and achieved level of engagement, activity, and/or disengagement across different life dimensions. These *consequences* manifest themselves as patterns of ageing and behaviour reflected in c*oping outcomes*, *trajectories* of ageing, and *meaning of life and death.* They are the result of interacting objective (physical and cognitive well-being) and subjective (psychological and spiritual well-being) outcomes of health within one's contextual environment across physical, social, economic, and health systems.

**Fig. 3:**
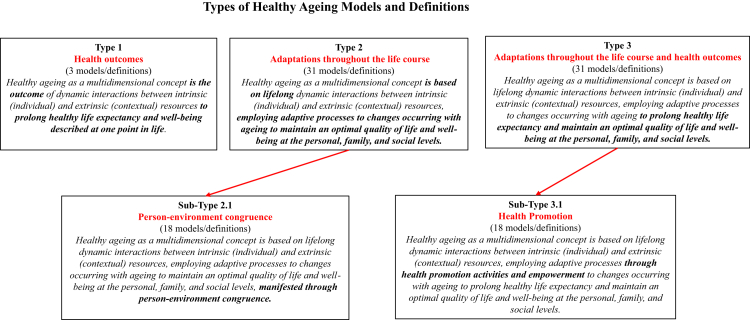
**Types of healthy ageing models and definitions**.

*Types of HA models and definitions*: Based on the identified antecedents, attributes, and consequences describing the processes and outcomes, three *types and two subtypes* of models and definitions emerged (Fig. 3):

*Type 1 Health outcomes* Cognitive, physical, and psychological health are the core elements of these models and lifelong processes are not explicitly described.^41^^,^^57^^,^^65^

*Type 2 Adaptations throughout the lifecourse* These models define the concept as a pathway of adaptative, compensatory, and/or development processes to changing environments and goals, and to gains and losses with ageing across multiple life dimensions. Health problems are not explicit (e.g. maintaining cognitive functioning is omitted but can be implied as cognition underlies biological processes etc.). Instead, adaptation processes are central and determine the ageing pattern based on the adjusted life in old age. This adjustment is described through a set of objective and subjective outcomes such as positive functioning and well-being, meaningful relationships and life satisfaction, optimal quality of life and autonomy, productivity, and identity development.^3^^,^^28^^,^^31^^,^^38^, ^39^, ^40^^,^^42^^,^^44^, ^45^, ^46^^,^^49^, ^50^, ^51^, ^52^, ^53^^,^^55^^,^^58^^,^^61^^,^^64^^,^^66^, ^67^, ^68^^,^^70^^,^^72^^,^^79^, ^80^, ^81^

*Sub-type 2.1 Person-environment congruence* The person-environment fit, also referred to as congruence or mastery, is an explicit contextual factor of adaptation that depends on life-acquired resourcefulness, resilience, and proactivity. Individuals create adjusted spaces, ecologies, or subcultures to age well in adequate environments. This adequacy involves demographic, social, cultural, political, economic, psychological, cognitive, physical, and spiritual dimensions.^31^^,^^39^^,^^40^^,^^42^^,^^44^, ^45^, ^46^^,^^52^^,^^53^^,^^55^^,^^58^^,^^64^^,^^66^, ^67^, ^68^^,^^70^^,^^72^^,^^80^ Most models build on clear theoretical frameworks: activity, disengagement, aged subculture, lifespan development, social construction, value orientations, motivational, and ecology theories. The outcomes describe belonging and ageing well in the environment and development of identity, self-conception, life satisfaction, meaning, spirituality, goal attainment, quality of life, positive functioning, activity, and productivity.

*Type 3 Adaptations throughout the life course and health outcomes* They combine types 1 and 2 approaches by focusing on adaptation and optimization to strengthen resilience while emphasizing the importance of maintaining health across physical and cognitive dimensions. Subjective well-being is equally highlighted to include life satisfaction, goal attainment, generativity, and engagement across personal, professional, and social dimensions.^4^^,^^5^^,^^26^^,^^27^^,^^29^^,^^30^^,^^32^, ^33^, ^34^, ^35^, ^36^, ^37^^,^^43^^,^^47^^,^^48^^,^^54^^,^^56^^,^^57^^,^^59^^,^^60^^,^^62^^,^^63^^,^^69^^,^^71^^,^^73^, ^74^, ^75^, ^76^, ^77^, ^78^ Rowe and Kahn's model constitutes the base for some of these models: proposing additional subjective dimensions such as spirituality and personal ageing experience,^76^ adapting it to LMICs by adding an objective environmental dimension,^69^ or proposing a comprehensive concept for practical strategies to ageing with disabilities.^30^

*Sub-type 3.1 Health promotion* Health promotion and empowerment activities are central to strengthening resilience and improving reserve capacities to limitations with ageing across cognitive, physical, psychological, cultural, demographic, economic, social, and spiritual dimensions.^4^^,^^5^^,^^26^^,^^27^^,^^32^, ^33^, ^34^, ^35^, ^36^, ^37^^,^^43^^,^^47^^,^^59^^,^^62^^,^^63^^,^^69^^,^^71^^,^^76^ These activities were framed in different ways across models. Some focused on community gerontology through a practical capacity-building framework for health promotion and engagement of older adults to maintain physical, mental, social, and subjective well-being and life satisfaction.^43^ Others adopted a practical health behaviour change approach for achieving self-efficacy and personal goals, tackled physical activity and personal predispositions in ageing to achieve subjective well-being, or highlighted the importance of adequate opportunities for optimal health and quality of life to strengthen resilience from childhood.^27^^,^^59^^,^^63^ Moreover, certain models built on theories of activity, disengagement, gero-transcendence, or the WHO's active ageing framework for health promotion for HA.^5^^,^^26^^,^^34^

## Discussion

Our study shows that HA has been recurrently and heterogeneously conceptualized in the literature, mostly from developed countries. Notwithstanding, there is consensus that HA depends on personal characteristics, resources, goals, and context-specific factors across subjective and objective dimensions: cognitive, physical, psychological, social, environmental, political, cultural, economic, demographic, and spiritual. HA can be defined based on two dominant approaches, separately or combined: (1) through health outcomes across cognitive, physical, social, and psychological dimensions, mainly depicted as the absence of disease and disability at the individual level and compression of morbidity and mortality at the population level, and (2) through developmental adaptation processes of lifelong, dynamic person-environment interactions to changes accompanying ageing across many dimensions. HA can be further defined based on congruence with the environment or health promotion and empowerment.

Similar to our findings, Wahl et al. classified the HA models according to objective and subjective criteria and adaptive mechanisms.^19^ Other reviews built the case for broader environmental, developmental, and adaptive approaches, some highlighting disability and the continuous reconstruction of the self given changes with ageing.^20^^,^^21^^,^^82^, ^83^, ^84^ More than three decades ago, Ryff compared HA conceptualizations and called for integrating lifespan development theories in psychology to strengthen the theoretical frameworks for each dimension.^83^ Based on our review, models from the past 20 years build upon earlier ageing theories, lifespan, and psychological development, mainly as adaptations of previously proposed models to certain contexts and populations.

Our findings indicate that one comprehensive theoretical model or monistic definition of HA, even if desired, is practically challenging. The emphasis on person-context interactions and the constantly evolving understanding of subjective and objective dimensions in HA underlies this impracticality. For instance, certain psychological/behavioural constructs are today widely accepted as sociocultural responses to life events rather than mere biological individual manifestations.^85^ This point becomes more evident when our findings are compared with the WHO HA definition. Despite the overall alignment as a lifelong process that builds on intrinsic capacities, functional abilities, and the interaction with the environment, there seems to be an overlap between domains in the WHO definition which requires further distinction to enable harmonization of operationalizations. For instance, the contribution to society that WHO classifies as a functional ability dimension overlaps with the environment dimension, and would be considered a social dimension based on our synthesis. Furthermore, there seems to be an elusiveness and complexity intrinsic to the conceptualization of health, which is foundational to HA. Haverkamp et al. argue that health is a family of diverse “thick concepts” that, although interrelated, cannot be unified under one single concept.^14^ In analyzing the social gerontology critiques of Rowe and Kahn's model, Martinson et al. found that when many criteria are used to generate an inclusive model, the result was sometimes a more exclusionary one.^18^ Consequently, the conceptual variety and multidimensionality we found can, to a considerable extent, explain the heterogeneity found in HA operational definitions. It renders the comparison of findings, their validity, applicability, and impact on healthcare decisions and policies quite challenging.^6^^,^^7^^,^^86^

This review provides a rationale for a dimension, population, and context-specific conceptual guidance that can bridge the gap between theory and practice and simplify HA operationalizations across dimensions. Of note are evident gaps for conceptualizations which are specific to gender, disability, and ethnicity as well as to LMICs. Our findings also provide a basis to critically reflect and rethink existing social and healthcare systems, which can enable HA by transitioning from a negative focus on ageism and disease management to a more positive, adaptive, and supportive context-specific approach. This could be achieved, for example, through priority-guided interventions and equitable opportunities for health promotion throughout the life course to strengthen baseline reserve capacities. Furthermore, providing adequate resources and supportive environments for individuals to prosper and age healthily based on their goals, network, and priorities are quintessential contextual factors.

It also is important to note that concept, model, definition, and theory are terms used interchangeably in these conceptualizations. If we intend to develop operational definitions that are theoretically solid and practically useful, there is an inevitable need to differentiate between terms in theorization, research, and practice, and adopt theory development methods to this field. Furthermore, the evolution of conceptualizations of HA is evident in the theories and the choice of terms across models, most of which were used in our search strategy and fulfilled our inclusion criteria for the models they describe. These reflect the contexts and times in which they have been produced and carry a normative approach to the concept of healthy ageing that evolves with the evolution of terms.^20^ Disengagement, activity, adjustment, and successful ageing have dominated most of the earlier literature originating from the USA, paralleling a predominant concern of youth unemployment and retirement policies and convey a certain judgment of ageing, which is considerably based on the level of social engagement and productivity. At the turn of the century, active, resilient, and HA became more frequent in Europe.^12^ This evolution coincides with the emerging concerns around the demographical changes and the WHO frameworks of active and HA that shifted the discourse to personality, agency, health promotion, and the context in developing resilience and maintaining a person-environment fit. The models as reflected by their describing terms represent an evolution from adaptation only or disease-specific to a more balanced, holistic, and context-specific approach combining objective and subjective dimensions. Furthermore, the more recent HA models and their normative terms seem primarily geared toward public utilitarian functions such as sustainability, productivity, and success. In other words, HA is somehow linked to economic improvement, which is essential. Thus, it's equally important to understand what ageing populations most value in life. Beyond cognitive and physical functional outcomes and self-caring abilities, key aspects of wellbeing such as friendship, socialization, sexuality, and love shall be better understood and integrated into the literature.

The findings of this review should be interpreted in light of certain limitations. We excluded book chapters and non-peer-reviewed articles which means that certain concepts and governmental/non-governmental reports on theoretical frameworks have not been included. Furthermore, our adopted definitions for dimensions and their classifications as subjective or objective might differ from other definitions used in papers and might appear more numerous in terms of dimensions than those initially described by authors. This adaptation was necessary to harmonize the heterogeneity in dimensions for comparative purposes. Furthermore, the conceptual nature of this systematic review might have introduced subjectivity, mitigated in the discussions of the thematic analysis and types with the authors. By describing the process in detail and developing a glossary of terms for dimensions, we aimed to produce a reproducible classification. Finally, our objective was not to conduct a review of mechanisms of ageing or developmental psychology and stage theories across the life course. We also did not aim to do a concept analysis *per se* to identify all possible empirical referents and model cases of HA as such approaches have been adopted before. Our focus was on the theory rather than the empirical use of HA concepts.

This paper provides a comprehensive overview of theoretical HA models. Our analysis identified the terms, dimensions, characteristics, antecedents, and consequences of HA conceptualizations. Our classification of models is based on the emphasis on health outcomes and/or adaptation processes, also highlighting person-environment congruence or health promotion across the life course approaches. It has become clear that a monistic model or definition of HA cannot practically accommodate the heterogeneous concept across different populations, dimensions, and contexts. Our type classification provides a basis for further harmonizing the use of conceptual terms and dimensions as well as theoretical grounds specific to HA dimensions that can guide research and comparisons of empirical findings. This would inform social and health policies enabling HA for different contexts and populations.

## Contributors

OHF and MM contributed to the conceptualization and design of the review protocol and registration. MM, OHF, and BM designed the search strategy run across all databases by BM. MM screened all articles by title, abstract, and full text in pairs with FK, ZMRD, OPE, MG, OI, JB, and FW. OHF and KS supervised MM, who conducted the data extraction, analysis, and classification of models by types, which were iteratively revised and discussed until agreement was reached with OHF and KS. MM wrote the original draft of the manuscript. All authors reviewed, edited, and approved the manuscript.

## Data sharing statement

Data inventories are provided as supplementary material.

## Declaration of interests

The authors declare no competing interests.

## Appendix A Supplementary data

Captions for Supplementary Material

Supplementary material_Revised_Submitted_eClinMedDec22
