# Supplementary material for: Concepts and definitions of healthy ageing: a systematic review and synthesis of theoretical models
Source: eClinicalMedicine. 2023 Jan 12;56:101821. doi: 10.1016/j.eclinm.2022.101821 (PMC9852292; doi:10.1016/j.eclinm.2022.101821)
Supplement: Captions for Supplementary Material [file mmc1.docx]

**Supplement 1. Full search strategy per database**

**Supplement 2. List of studies excluded at full text screening with reasons
Supplement 3. Summary of findings**

**Supplement 4. Inventory: Healthy ageing concept: antecedents, consequences, and attributes**
