# Supplementary material for: Concepts and definitions of healthy ageing: a systematic review and synthesis of theoretical models
Source: eClinicalMedicine. 2023 Jan 12;56:101821. doi: 10.1016/j.eclinm.2022.101821 (PMC9852292; doi:10.1016/j.eclinm.2022.101821)
Supplement: Supplementary material_Revised_Submitted_eClinMedDec22 [file mmc2.pdf]

Contents

Supplement 1. Full search strategy per database 2

Supplement 2. List of studies excluded at full text screening with reasons 3

Supplement 3. Summary of findings 8

Supplement 4. Inventory: Healthy ageing concept: antecedents, consequences, and attributes 12

Supplementary Material

Supplement 1. Full search strategy per database

Database	Search strategy
Embase.com	('healthy aging'/de OR 'successful aging'/de OR (((healthy OR healthful OR healthier OR successful OR optimal OR well OR active OR robust OR positive OR productive OR effective OR phenotype*) NEAR/1 (ageing* OR aging*))):ab,ti) AND ((definition* OR construct* OR facet* OR domain* OR model* OR theor* OR concept* OR dimension* OR score* OR index* OR indices OR scal*):ab,ti) NOT ([animals]/lim NOT [humans]/lim) NOT ([Conference Abstract]/lim OR [Letter]/lim OR [Note]/lim OR [Editorial]/lim OR [Review]/lim OR [Preprint]/lim)
Medline ALL (Ovid)	(Healthy Aging/ OR (((healthy OR healthful OR successful OR optimal OR well OR active OR robust OR positive OR productive OR effective OR phenotype*) adj1 (ageing* OR aging*))).ab,ti.) AND ((definition* OR construct* OR facet* OR domain* OR model* OR theor* OR concept* OR dimension* OR score* OR index* OR indices OR scal*).ab,ti.) NOT (exp animals/ NOT humans/) NOT (letter OR news OR comment OR editorial OR congress OR abstracts OR review OR preprint).pt.
Cochrane CENTRAL register of controlled trials	((((healthy OR healthful OR healthier OR successful OR optimal OR well OR good OR active OR robust OR positive OR productive OR effective OR phenotype*) NEAR/1 (ageing* OR aging*))):ab,ti) AND ((definition* OR construct* OR facet* OR domain* OR model* OR theor* OR concept* OR dimension* OR score* OR index* OR indices OR scal*):ab,ti)
CINAHL (EBSCOhost)	(MH "Healthy Aging" OR (((healthy OR healthful OR successful OR optimal OR well OR active OR robust OR positive OR productive OR effective OR phenotype*) N1 (ageing* OR aging*)))) AND (definition* OR construct* OR facet* OR domain* OR model OR models OR theor* OR concept* OR dimension* OR score* OR index* OR indices OR scal*) NOT (MH animals+ NOT MH humans+) NOT PT (letter OR commentary OR response OR editorial OR abstract OR review)
PsycINFO (OvidSP)	(healthy aging/ OR (((healthy OR healthful OR successful OR optimal OR well OR active OR robust OR positive OR productive OR effective OR phenotype*) adj1 (ageing* OR aging*))).ab,ti.) AND ((definition* OR construct* OR facet* OR domain* OR model* OR theor* OR concept* OR dimension* OR score* OR index* OR indices OR scal*).ab,ti.) NOT (exp animals/ NOT humans/) NOT (letter OR comment OR editorial OR abstract OR review*).dt
Web of Science Core Collection	TS=((((healthy OR healthful OR successful* OR optimal* OR well OR active OR robust OR positive OR productive OR effective OR phenotype*) NEAR/0 ("ageing*" OR "aging*")))) AND TS=(definition* OR construct* OR facet* OR domain* OR model OR models OR theor* OR concept* OR dimension* OR score* OR index OR indices OR scal*) NOT TS=((animal* OR plant* OR rats OR mice OR pigs OR murine OR primate* OR monkey*) NOT (human* OR patient*)) AND DT=(article OR early access)

Supplement 2. List of studies excluded at full text screening with reasons

Author	Year	Title	Reason for exclusion (n=84)
Pincus	1967	Toward a Developmental View of Aging for Social Work	Review or critique without a clear model that fits criteria for inclusion
Ryff	1982	Successful Aging: A Developmental Approach	Review or critique without a clear model that fits criteria for inclusion
Johnson	1995	Ageing well in contemporary society	Review or critique without a clear model that fits criteria for inclusion
Minkler et al	2002	Successful ageing	Review or critique without a clear model that fits criteria for inclusion
Aguerre et al	2003	Successful aging: Theory, research and clinical applications	Review or critique without a clear model that fits criteria for inclusion
Wiesman et al	2004	Salutogenesis in old age	Review or critique without a clear model that fits criteria for inclusion
Chapman	2005	Theorizing about aging well: Constructing a narrative	Review or critique without a clear model that fits criteria for inclusion
Rohr et al	2009	Aging well together-A mini-review	Review or critique without a clear model that fits criteria for inclusion
Carrascosa-Gil et al	2010	Successful aging: a holistic perspective	Review or critique without a clear model that fits criteria for inclusion
Fernandez-Ballesteros	2011	Positive ageing: Objective, subjective, and combined outcomes	Review or critique without a clear model that fits criteria for inclusion
Friedman et al	2012	Theoretical perspectives: A biopsychosocial approach to positive aging	Review or critique without a clear model that fits criteria for inclusion
Boudiny	2013	Active ageing: from empty rhetoric to effective policy tool	Review or critique without a clear model that fits criteria for inclusion
Fereshtehnejad eta l	2014	Active Aging for Individuals with Parkinson's Disease: Definitions, Literature Review, and Models	Review or critique without a clear model that fits criteria for inclusion
Ji H	2015	Successful aging in the United States and China: a theoretical basis to guide nursing research, practice, and policy	Review or critique without a clear model that fits criteria for inclusion
Midlarsky E	2015	Prosocial behavior in late life	Review or critique without a clear model that fits criteria for inclusion
Zacher H	2015	The Importance of a Precise Definition, Comprehensive Model, and Critical Discussion of Successful Aging at Work	Review or critique without a clear model that fits criteria for inclusion
Hanson et al	2016	Developmental aspects of a life course approach to healthy ageing	Review or critique without a clear model that fits criteria for inclusion
Repeprmund et al	2016	Successful ageing for people with an intellectual disability	Review or critique without a clear model that fits criteria for inclusion
Geard et al	2017	Masters Athletes: Exemplars of Successful Aging?	Review or critique without a clear model that fits criteria for inclusion
Cosco et al	2018	Psychosocial aspects of successful ageing and resilience: Critique, integration and implications	Review or critique without a clear model that fits criteria for inclusion
Serrano	2018	Active ageing: A paradigm for understanding and governing	Review or critique without a clear model that fits criteria for inclusion
Attila	2019	A pozitív öregedés pszichológiai forrásai	Review or critique without a clear model that fits criteria for inclusion
Quigley et al	2022	Aging Well for Indigenous Peoples: A Scoping Review	Review or critique without a clear model that fits criteria for inclusion
Pocock et al	2022	Diverse approaches to conceptualising positive ageing: A scoping review	Review or critique without a clear model that fits criteria for inclusion

Johansson	1987	Declining resources in old age: Theoretical aspects and implications for an old-age policy	Book/chapter/dissertation
Featherman et al	1990	Successful aging in a post-retired society	Book/chapter/dissertation
Thomae	1990	Stress, satisfaction, competence: Findings from the Bonn Longitudinal Study on Aging	Book/chapter/dissertation
Baltes	1992	Wisdom and successful aging	Book/chapter/dissertation
Brandtstadter	1993	Development, aging, and control: Empirical and theoretical issues	Book/chapter/dissertation
Dsouza	1993	The concept of active ageing	Book/chapter/dissertation
Midlarsky et al	1994	Altruism in later life	Book/chapter/dissertation
Kahana et al	1996	Conceptual and empirical advances in understanding aging well through proactive adaptation	Book/chapter/dissertation
De Oliveira	2002	Aging: Scientific versus folk theories	Book/chapter/dissertation
Guo	2007	Self-imposed activity limitation among community dwelling elders	Book/chapter/dissertation
Szanton et al	2010	The society-to-cells model of resilience in older adults	Book/chapter/dissertation
Maddox	1965	Fact and Artifact: Evidence Bearing on Disengagement Theory from the Duke Geriatrics Project	Design (Conference/empirical/validation/score/clinical/intervention frameworks/mathematical modeling/opinion/psychological stage development theory/other)
Band	1977	Continuity of life situationa nd succesfsul ageing (German)	Design (Conference/empirical/validation/score/clinical/intervention frameworks/mathematical modeling/opinion/psychological stage development theory/other)
Tempelman	1977	Successful ageing: A new learning theory viewpoint (Dutch)	Design (Conference/empirical/validation/score/clinical/intervention frameworks/mathematical modeling/opinion/psychological stage development theory/other)
Deshpande et al	1990	Non-Monotonic Ageing	Design (Conference/empirical/validation/score/clinical/intervention frameworks/mathematical modeling/opinion/psychological stage development theory/other)
Wacks	1994	Realizing our inner elder-child: Toward the possible human	Design (Conference/empirical/validation/score/clinical/intervention frameworks/mathematical modeling/opinion/psychological stage development theory/other)
Kerschner	1998	Productive ageing: a quality of life agenda	Design (Conference/empirical/validation/score/clinical/intervention frameworks/mathematical modeling/opinion/psychological stage development theory/other)
Nussbaum	2000	Message Production Across the Life Span: Communication and Aging	Design (Conference/empirical/validation/score/clinical/intervention frameworks/mathematical modeling/opinion/psychological stage development theory/other)
Parker et al	2001	Soldier and Family Wellness across the Life Course: A Developmental Model of Successful Aging, Spirituality, and Health Promotion, Part II	Design (Conference/empirical/validation/score/clinical/intervention frameworks/mathematical modeling/opinion/psychological stage development theory/other)
Fries	2002	Successful aging—an emerging paradigm of gerontology	Design (Conference/empirical/validation/score/clinical/intervention frameworks/mathematical modeling/opinion/psychological stage development theory/other)
Torres	2002	Relational Values and Ideas Regarding "Successful Aging"*	Design (Conference/empirical/validation/score/clinical/intervention frameworks/mathematical modeling/opinion/psychological stage development theory/other)

Torres	2003	A preliminary empirical test of a culturally-relevant theoretical framework for the study of successful aging	Design (Conference/empirical/validation/score/clinical/intervention frameworks/mathematical modeling/opinion/psychological stage development theory/other)
Bowling et al	2006	Which model of successful ageing should be used?Baseline study from a British longitudinal survey of ageing	Design (Conference/empirical/validation/score/clinical/intervention frameworks/mathematical modeling/opinion/psychological stage development theory/other)
Harris	2008	Another wrinkle in the debate about successful aging: The undervalued concept of resilience and the lived experience of dementia	Design (Conference/empirical/validation/score/clinical/intervention frameworks/mathematical modeling/opinion/psychological stage development theory/other)
Fernandez-Ballesteros et al	2010	The concept of 'ageing well' in ten Latin American and European countries	Design (Conference/empirical/validation/score/clinical/intervention frameworks/mathematical modeling/opinion/psychological stage development theory/other)
Swindel	2010	Indicators of "healthy aging" in older women (65-69 years of age). A data-mining approach based on prediction of long-term survival	Design (Conference/empirical/validation/score/clinical/intervention frameworks/mathematical modeling/opinion/psychological stage development theory/other)
Lee et al	2011	Aging successfully: A four-factor model	Design (Conference/empirical/validation/score/clinical/intervention frameworks/mathematical modeling/opinion/psychological stage development theory/other)
Lezwijn	2011	Healthy ageing in a salutogenic way: Building the HP 2.0 framework	Design (Conference/empirical/validation/score/clinical/intervention frameworks/mathematical modeling/opinion/psychological stage development theory/other)
Parslow	2011	Successful Aging: Development and Testing of a Multidimensional Model Using Data From a Large Sample of Older Australians	Design (Conference/empirical/validation/score/clinical/intervention frameworks/mathematical modeling/opinion/psychological stage development theory/other)
Cho et al	2012	The Older TheyAre, the Less Successful TheyBecome? Findings from the Georgia Centenarian Study	Design (Conference/empirical/validation/score/clinical/intervention frameworks/mathematical modeling/opinion/psychological stage development theory/other)
Doyle et al	2012	A model of successful ageing in British populations	Design (Conference/empirical/validation/score/clinical/intervention frameworks/mathematical modeling/opinion/psychological stage development theory/other)
Jeon	2012	Dynamics of Constructs in Successful Aging of Korean Elderly: Modified Rowe and Kahn's Model	Design (Conference/empirical/validation/score/clinical/intervention frameworks/mathematical modeling/opinion/psychological stage development theory/other)
Paul et al	2012	Active Ageing: An Empirical Approach to the WHO Model	Design (Conference/empirical/validation/score/clinical/intervention frameworks/mathematical modeling/opinion/psychological stage development theory/other)
Randall	2012	Successful aging: A psychosocial resources model for very old adults	Design (Conference/empirical/validation/score/clinical/intervention frameworks/mathematical modeling/opinion/psychological stage development theory/other)
Vahia et al	2012	Developing a dimensional model for successful cognitive and emotional aging	Design (Conference/empirical/validation/score/clinical/intervention frameworks/mathematical modeling/opinion/psychological stage development theory/other)

Woods et al	2012	Toward a positive aging phenotype for older women: observations from the women's health initiative	Design (Conference/empirical/validation/score/clinical/intervention frameworks/mathematical modeling/opinion/psychological stage development theory/other)
Cosco et al	2013	Deathless models of aging and the importance of acknowledging the dying process	Design (Conference/empirical/validation/score/clinical/intervention frameworks/mathematical modeling/opinion/psychological stage development theory/other)
Lamb	2014	Permanent personhood or meaningful decline? Toward a critical anthropology of successful aging	Design (Conference/empirical/validation/score/clinical/intervention frameworks/mathematical modeling/opinion/psychological stage development theory/other)
Pietrzak et al	2014	Successful aging among older veterans in the United States	Design (Conference/empirical/validation/score/clinical/intervention frameworks/mathematical modeling/opinion/psychological stage development theory/other)
Tam et al	2014	Understanding and theorizing the role of culture in the conceptualizations of successful aging and lifelong learning	Design (Conference/empirical/validation/score/clinical/intervention frameworks/mathematical modeling/opinion/psychological stage development theory/other)
Benberin et al	2015	Medical social modeling technologies for active ageing in Kazakhstan	Design (Conference/empirical/validation/score/clinical/intervention frameworks/mathematical modeling/opinion/psychological stage development theory/other)
Cho et al	2014	Successful Aging and Subjective Well-Being Among Oldest-Old Adults	Design (Conference/empirical/validation/score/clinical/intervention frameworks/mathematical modeling/opinion/psychological stage development theory/other)
Oliver et al	2016	Validación de un modelo explicativo del proceso de envejecer con éxito a partir de aspectos psicológicos, físicos, relacionales y de ocio (Spanish)	Design (Conference/empirical/validation/score/clinical/intervention frameworks/mathematical modeling/opinion/psychological stage development theory/other)
Peisah	2016	Successful ageing for psychiatrists	Design (Conference/empirical/validation/score/clinical/intervention frameworks/mathematical modeling/opinion/psychological stage development theory/other)
Gawcett et al	2017	Optimal Aging: A Neuman Systems Model Perspective	Design (Conference/empirical/validation/score/clinical/intervention frameworks/mathematical modeling/opinion/psychological stage development theory/other)
Cesari et al	2018	Evidence for the Domains Supporting the Construct of Intrinsic Capacity	Design (Conference/empirical/validation/score/clinical/intervention frameworks/mathematical modeling/opinion/psychological stage development theory/other)
Gore et al	2018	New horizons in the compression of functional decline	Design (Conference/empirical/validation/score/clinical/intervention frameworks/mathematical modeling/opinion/psychological stage development theory/other)
Ko et al	2019	Childhood conditions and productive aging in China	Design (Conference/empirical/validation/score/clinical/intervention frameworks/mathematical modeling/opinion/psychological stage development theory/other)
McGarrigle et al	2019	Cognitive Reserve Capacity: Exploring and Validating a Theoretical Model in Healthy Ageing	Design (Conference/empirical/validation/score/clinical/intervention frameworks/mathematical modeling/opinion/psychological stage development theory/other)

Sebert et al	2019	Cohort Profile: The DynaHEALTH consortium – a European consortium for a life-course bio-psychosocial model of healthy ageing of glucose homeostasis	Design (Conference/empirical/validation/score/clinical/intervention frameworks/mathematical modeling/opinion/psychological stage development theory/other)
صفورا دري*	2019	مفهوم سالمندي فعال در يه ابتلا به د ت يک مقاله کيفی (Farsi)	Design (Conference/empirical/validation/score/clinical/intervention frameworks/mathematical modeling/opinion/psychological stage development theory/other)
Lucena et al	2020	Multidimensional model of successful aging and nursing terminologies:<br>similarities for use in the clinical practice	Design (Conference/empirical/validation/score/clinical/intervention frameworks/mathematical modeling/opinion/psychological stage development theory/other)
Mendoza-Núñez et al	2018	Modelo comunitario de envejecimiento saludable enmarcado en la resiliencia y la generatividad (Spanish)	Duplicate
Chandler	1948	Cicer's ideal old man	Citation tracking (exclude, design (review/stage theory, opinion/other)
Rose	1962	The Subculture of the Aging: A Topic for Sociological Research1	Citation tracking (exclude, design (review/stage theory, opinion/other)
Robert	1974	Successful Aging and the Role of the Life Review*	Citation tracking (exclude, design (review/stage theory, opinion/other)
Ryff	1982	Successful Aging: A Developmental Approach	Citation tracking (exclude, design (review/stage theory, opinion/other)
Tornstam	1989	Gero-transcendence: A reformulation of the disengagement theory	Citation tracking (exclude, design (review/stage theory, opinion/other)
Baltes	1993	The Aging Mind: Potential and Limits	Citation tracking (exclude, design (review/stage theory, opinion/other)
Antonovsky	1996	The salutogenic model as a theory to guide health promotion	Citation tracking (exclude, design (review/stage theory, opinion/other)

Supplement 3. Summary of findings

Summary of findings (Supplementary Material)				
	**Country of publication (corresponding author)**	**Year**	**# of papers**	**% from papers**
	Brazil	2021	1	2%
	Canada	2006	1	2%
	China	2013	1	2%
	Germany	1993-2017	6	10%
	Italy	2021	1	2%
	Jamaica	2016	1	2%
	Mexico	2017-2022	3	5%
	New Zealand	2015	1	2%
	Philippines	2022	1	2%
	Spain	2012	1	2%
	Sweden	1999,2015	2	3%
	Switzerland	2003,2021	2	3%
	Taiwan	2013	1	2%
	The Netherlands	1998-2020	5	8%
	UK	2001	1	2%
	USA	1960-2019	31	53%
**Sub-total (papers)**	**16**	**1960-2022**	**59**	**100%**
**Normative Terms (59 papers, 65 models)**			**# of models**	**% from models**
Active Ageing	Switzerland, The Netherlands, Jamaica, Mexico	2003-2017	4	6%
Adjustment to ageing	USA	1966,1982	2	3%
Ageing (normative approach)	USA	1972-1999	3	5%
Ageing well	USA, Canada, Germany	1991,2006,2012	5	8%
Ageing theories	USA	1960,1972,199	3	5%
Compression of morbidity (normative to ageing)	USA	1980	1	2%
Graceful ageing	Taiwan	2013	1	2%
Healthy ageing, Child health in healthy ageing	Italy, Brazil, Switzerland, Mexico, USA, The Netherlands	2005-2021	8	12%
Mindful Sustainable Ageing	Sweden	2015	1	2%

Positive ageing	USA	2011	1	2%
Productive ageing/engagement	China, USA, Mexico	2013,2017,2022	3	5%
Resilient/optimal ageing	Germany, USA	1993,2014	2	3%
Successful ageing	USA, UK, Netherlands, Sweden, Spain, Germany, New Zealand, Philippines	1961-2022	34	52%
of which	USA	1961-2019	18	53%
	UK	2001	1	3%
	The Netherlands	1998,2015,2020	3	9%
	Sweden	1999	1	3%
	Spain	2012	1	3%
	Germany	1996-2017	4	12%
	New Zealand	2015	1	3%
	Philippines	2022	1	3%
**Total number of terms used**	12			
**Number of articles (models) included**	59 (61 models, 4 derived definitions)			
**Language**	English			
**Years of publication**		1960 to 2022		
**Models/theories in specific contexts/groups**	Out of 65 models	15	23%	
	Chinese/Americans populations	2	13%	
	People living with HIV	2	13%	
	Lesbian, Gay, Bisexual people	3	20%	
	People in LMICs	1	7%	
	Child health in healthy agieng	1	7%	
	People ageing at work	5	33%	
	People with care needs	1	7%	
**Sub-total (models)**		**15**	**100%**	
**Paradigm**				
	Theoretical or philosophical	65	100%	

	Practical outlook	12	18%	
	Empirically validated original data	6	9%	
	Derived definitions	1 theory	4 definitions	
	Theory with definition or description	8	12%	
	Middle-range theories	3	38%	
	Concept analyses	7	11%	
	Based on other conceptual frameworks	45	69%	
**Source (Papers)**				
	Original/secondary source articles (not forward search)	45	76%	
	Forward search	7	12%	
	Concept analyses	7	12%	
**Sub-total (papers)**		**59**	**100%**	
**Approach (models)**				
	Lifecourse	62	95%	
	Outcome	3	5%	
**Sub-total (models)**		**65**	**100%**	
**Dimensions in models/theories/definitions (several models could be in same article) (out of 65)**	2 to 9			
Cognitive	62	95%		
Cultural	8	12%		
Demographic	4	6%		
Economic	13	20%		
Environmental	19	29%		
Political	6	9%		
Psychological	53	82%		
Physical	49	75%		
Social	49	75%		
Spiritual	16	25%		

**Dimensions number per model**	**Number of dimensions**	**Number of models**	%	
	2	8	12%	
	3	11	17%	
	4	19	29%	
	5	16	25%	
	6	5	8%	
	7	3	5%	
	8	1	2%	
	9	2	3%	
**Sub-total (models)**		**65**	**100%**	
**Subjective or objective dimensions**				
	Objective	8	12%	
	Subjective, Objective	57	88%	
**Sub-total (models)**		**65**	**100%**	

Supplement
3_HA%20Concept%2

Supplement 4. Inventory: Healthy ageing concept: antecedents, consequences, and attributes

Antecedents

Intrinsic Factors

Socio-demography (biography) life narrative, life course, personal characteristics ;
Goals & values s pirituality, health -faith synergies, personal health system ;
Psychological/ behavioural characteristics psychological reserves, intrapsychic and personality (optimism, self -efficacy, motivation), emotions, behaviour;
Physical capacities r eserve capacity , health and vigor, growth rate (peak capacity) and rate of senescence (peak time), illness (normative stressor) & comorbidities;
Cognitive capacities r eserve capacity, cognitive mechanics and pragmatics, losses in capacities, comorbidities ;

Temporal context:

age reached, remaining time, perception of time

Individual -environment interactions , communication ,cooperation

Extrinsic Factors

Environment latent resources/strategies, physical (built & natural) environment, predictable external events/stressors/stimuli, systems in place, stakeholders, constraints, opportunities, technology ;
Social system societal provisions/resources, social policies, social engagement/support, family context/support, changes in social environment ;
Political system, government laws , public policies ;
Culture norms/values, stereotypes of ageing, context (historical)
Economic/organizational systems l egislations, pension system, labor market, financial adequacy/resources, facilities, affordable transport ;
Educational & health systems promotion of health, activity resources/constraints, access to information, focused interventions, healthcare ;

Attributes

Dimensions

physical, cognitive, psychological, environmental ,political, economic, cultural, social -,spiritual dimensions ;

Dynamic interactions, Interplay of individual -contextual dimensions across
macro ,meso, micro, and chronosystems

Processes along the life course

Lifespan development vs ageing dynamics belonging & agency, generativity;
Selection, Optimization, Compensation processes preventive and corrective adaptations (accommodative, assimilative, defensive), substitution/ transformation processes, self-regulation processes (proactive and adaptive (dis) engagement and coping, goal setting and pursuit), transcendence processes and shift in perspective, adaptive behaviour over the life course (integrated, compensatory, compromised processes) , mainly:

- **Psychological/cognitive processes** cognitive adaptive mechanisms, ecologies of ageing processes linking affect, efficacy, and communication, acceptance/desire for disengagement, growth (develop one's potential), internal locus for behavioural regulation/autonomous functioning/evaluation, resistance/resilience /deliverance from convention & enculturation, pursuit of emotional goals, behavioural efforts of coping, mindfulness practices (shifting to inward life), development and adaptive regulation of the ageing self, ability to choose or create environments suitable to one's psychic conditions, substitution of fluid abilities with crystallized knowledge, restoration of deficits, education ;
- **Physical Processes** strengthening organ reserve, shifting premature organ dysfunction, adaptation of functional performance mechanisms , process of optimizing opportunities for health, participation and security ;
- **Socio-environmental processes** interaction of age and moderating variables at work, access to friendly healthcare services (populations LGB,HIV, special needs) ;

Consequences

Healthy lifespan
Objective and Subjective Outcomes

Physical functioning & well -being optimal functioning, biological health outcomes, longevity ;
Cognitive health optimal cognitive functioning, neurocognitive health, autonomy ;

Subjective well-being
Identity & self, self - assessed quality of life, spiritual ;

Person-Environment fit
Economic system *labor policies & work outcomes, occupational health*
Health systems *healthcare, health promotion and prevention, capacity strengthening*
Social *system social well -being, relationships, generativity, addressing the impact of life changes, participation, belonging, family/friend well -being*
Physical environment *maintaining home and living space, animal companionship, age -friendly environments*

Patterns of ageing (behaviour)

Successful coping outcomes with age-related deficits successfully adapt/adjust/engage (gradual, no sudden collapse), substitute activities, disengage socially and emotionally, balance activity and disengagement, overcome the developmental adversity, reduce risk of adverse events and enhance resilience in their presence, maintain consistency with previous life, maintain role/active old age, maintain productive engagement and work, find the intersection between value and attainability with changing situation, facilitate identification of goals and moving towards them, be self-sufficient, recover positively & sustain health adjustment, accept deficit (gratitude), accept help from others, possess a positive spirituality ;
Trajectories pathological, normal, optimal ageing; stereotypical, passing, affirmative ; progressive change, stability, regressive change ; restricted but effective life; first/second/third order criteria of success over the life course; healthy lifestyle, active living; balanced integration of useful traits; socially acceptable ageing in the contextual realm;
Way / meaning of life and death pattern of meaningful existence (making sense of the present in terms of the past), purpose & life course perspective for goals and health trajectory, ageing experience & reflection on life aspects (past and present, and future life plan), value orientations & subject-object meaning making, death meaning and acceptance ;
